# Supplementary material for: Pyruvate Dehydrogenase Kinase 1 inhibition mediated oxidative phosphorylation enhancement in cartilage promotes osteoarthritis progression
Source: BMC Musculoskelet Disord. 2023 Jul 20;24:597. doi: 10.1186/s12891-023-06585-6 (PMC10357736; doi:10.1186/s12891-023-06585-6)
Supplement: Supplementary file 1 — Additional file 1. [file 12891_2023_6585_MOESM1_ESM.zip › BP.Gsea.1653623667859/GOBP_NUCLEOSOME_MOBILIZATION.html]

Details for gene set GOBP\_NUCLEOSOME\_MOBILIZATION[GSEA]

|  || Dataset | 55457\_collapsed\_to\_symbols.cls.cls #OA\_versus\_normal.cls.cls #OA\_versus\_normal\_repos |
| Phenotype | cls.cls#OA\_versus\_normal\_repos |
| Upregulated in class | normal |
| GeneSet | GOBP\_NUCLEOSOME\_MOBILIZATION |
| Enrichment Score (ES) | -0.6074759 |
| Normalized Enrichment Score (NES) | -1.7532749 |
| Nominal p-value | 0.007380074 |
| FDR q-value | 0.11600393 |
| FWER p-Value | 0.998 |
Table: GSEA Results Summary

  

Fig 1: Enrichment plot: GOBP\_NUCLEOSOME\_MOBILIZATION      
 Profile of the Running ES Score & Positions of GeneSet Members on the Rank Ordered List

  

| SYMBOL | TITLE | RANK IN GENE LIST | RANK METRIC SCORE | RUNNING ES | CORE ENRICHMENT || 1 | UCHL5 | ubiquitin C-terminal hydrolase L5 [Source:HGNC Symbol;Acc:HGNC:19678] | 1031 | 0.511 | -0.0018 | No |
| 2 | RUVBL2 | RuvB like AAA ATPase 2 [Source:HGNC Symbol;Acc:HGNC:10475] | 2778 | 0.272 | -0.0957 | No |
| 3 | TFPT | TCF3 fusion partner [Source:HGNC Symbol;Acc:HGNC:13630] | 3226 | 0.228 | -0.0955 | No |
| 4 | POLE3 | "DNA polymerase epsilon 3, accessory subunit [Source:HGNC Symbol;Acc:HGNC:13546]" | 3934 | 0.162 | -0.1256 | No |
| 5 | MCRS1 | microspherule protein 1 [Source:HGNC Symbol;Acc:HGNC:6960] | 4286 | 0.129 | -0.1332 | No |
| 6 | ARID1A | AT-rich interaction domain 1A [Source:HGNC Symbol;Acc:HGNC:11110] | 5735 | 0.013 | -0.2436 | No |
| 7 | RUVBL1 | RuvB like AAA ATPase 1 [Source:HGNC Symbol;Acc:HGNC:10474] | 7477 | -0.116 | -0.3610 | No |
| 8 | ACTR5 | actin related protein 5 [Source:HGNC Symbol;Acc:HGNC:14671] | 10654 | -0.388 | -0.5481 | Yes |
| 9 | ACTR8 | actin related protein 8 [Source:HGNC Symbol;Acc:HGNC:14672] | 10749 | -0.399 | -0.4943 | Yes |
| 10 | NFRKB | nuclear factor related to kappaB binding protein [Source:HGNC Symbol;Acc:HGNC:7802] | 10758 | -0.399 | -0.4338 | Yes |
| 11 | ACTL6A | actin like 6A [Source:HGNC Symbol;Acc:HGNC:24124] | 10821 | -0.407 | -0.3763 | Yes |
| 12 | INO80D | INO80 complex subunit D [Source:HGNC Symbol;Acc:HGNC:25997] | 11213 | -0.459 | -0.3364 | Yes |
| 13 | SMARCA5 | "SWI/SNF related, matrix associated, actin dependent regulator of chromatin, subfamily a, member 5 [Source:HGNC Symbol;Acc:HGNC:11101]" | 11838 | -0.557 | -0.2997 | Yes |
| 14 | BPTF | bromodomain PHD finger transcription factor [Source:HGNC Symbol;Acc:HGNC:3581] | 11870 | -0.565 | -0.2156 | Yes |
| 15 | INO80B | INO80 complex subunit B [Source:HGNC Symbol;Acc:HGNC:13324] | 12103 | -0.621 | -0.1386 | Yes |
| 16 | YY1 | YY1 transcription factor [Source:HGNC Symbol;Acc:HGNC:12856] | 12229 | -0.653 | -0.0483 | Yes |
| 17 | BAZ1A | bromodomain adjacent to zinc finger domain 1A [Source:HGNC Symbol;Acc:HGNC:960] | 12238 | -0.655 | 0.0514 | Yes |
Table: GSEA details [plain text format]

  

Fig 2: GOBP\_NUCLEOSOME\_MOBILIZATION      
 Blue-Pink O' Gram in the Space of the Analyzed GeneSet

  

Fig 3: GOBP\_NUCLEOSOME\_MOBILIZATION: Random ES distribution      
 Gene set null distribution of ES for **GOBP\_NUCLEOSOME\_MOBILIZATION**
